# Hybrid organic-inorganic coatings via electron transfer behaviour

**DOI:** 10.1038/s41598-017-07691-x

**Published:** 2017-08-01

**Authors:** Wail Al Zoubi, Ji Hoon Min, Young Gun Ko

**Affiliations:** Materials Electrochemistry Laboratory, School of Materials Science and Engineering, Yeungnam University, Gyeongsan, 38541 Republic of Korea

## Abstract

A novel method to functionalize the surface of inorganic coating by growing organic coating has been investigated based on microstructural interpretation, electrochemical assessment, and quantum chemical analysis. For this purpose, inorganic coating with magnesium aluminate, magnesium oxide, and titanium dioxide was prepared on magnesium alloy via plasma electrolytic oxidation (PEO), and, then, subsequent dip-coating method was used to tailor organic coating using diethyl-5-hydroxyisophthalate (DEIP) as organic molecules. The incorporation of TiO_2_ particles worked as a sealing agent to block the micro-defects which resulted mainly from the intense plasma sparks during PEO. In addition, such incorporation played an important role in enhancing the adhesion between inorganic and organic coatings. The use of DEIP as organic corrosion inhibitor resulted in a significant decrease in porosity of inorganic coating. Quantum chemical calculation was used to clarify the corrosion inhibition mechanism which was activated by introduction of DEIP. Thus, the electrochemical analysis based on potentiodynamic polarization and impedance spectroscopy tests in 3.5 wt% NaCl solution suggested that corrosion resistance of magnesium alloy sample was enhanced significantly due to a synergistic effect arising from the hybrid inorganic and organic coatings. This phenomenon was explained in relation to electron transfer behaviour between inorganic and organic coatings.

## Introduction

Magnesium and its alloys have been applied in a wide range of lightweight engineering applications, such as the automotive, mobile, and biomedical fields, due to their good mechanical reliability based on low density^1, 2^. However, one of the critical shortcomings in magnesium alloys was known to be impoverished surface resistance against corrosion even under mild environments because of their high chemical activity and high negative electrical potential^3–6^. Thus, surface modification techniques, such as conversion coating, electroplating, anodisation, plasma electrolytic oxidation (PEO), organic coating, and vapour-phase process, have been developed to overcome the surface matter^7–9^. Among these methods, PEO coating was reported to be an eco-friendly technique suitable for valve metallic materials in which protective inorganic coating formed on the metal substrate with an aid of plasma-enhanced electrochemical reactions^7^. It is, however, certain that inorganic coatings with high porosity might suffer from rapid electrochemical reactivity as the discharge channels and the connected micro-pores present in coating would provide short circuit path for plausible occurrence of pitting corrosion^10^.

Several attempts have been made to modify the morphologies and structures of coatings by optimizing the electrical parameter, electrolyte condition, post-treatment, etc. The effects of the external electrical parameter and the base electrolyte on the development of coating and the related electrochemical response have been documented. Regarding electrolyte additives^11^, the corrosion resistance of inorganic coating would be enhanced by incorporating inorganic nanoparticles, such as ZrO_2_, SiO_2_, and TiO_2_, which might produce a relatively compact structure of inorganic coating with low porosity^12^. Subsequent to PEO, dip-coating (DC) as one of the post-treatments performed chemically in a specific solution containing organic inhibitors turned out to be desirable for blocking the micro-defects in inorganic coating. Thus, a series combination of electrolyte modification and DC with organic compounds would lead to the inorganic-organic coatings which would exhibit desirable endurance even under the harsh corrosive environment. It is undoubtful that the reliable bonding strength between inorganic and organic coatings remained unresolved. In this study, hence, TiO_2_ nanoparticles with attractive inherent properties^13^ were selected as secondary substance in the electrolyte for the present PEO coating in order not only to improve the attachment of organic coating to inorganic coating, also to improve the corrosion properties of inorganic coating by electrophoretic incorporation of inorganic nanoparticles as aforementioned above.

Organic additives, such as tartaric acid (beverage additive) and 2.6-diamniopyridine (organic dye), adsorbed on the surface of coating were found to prevent the adsorption of chloride ions as well as to trigger the formation of protective oxide coating on the metal surface^14, 15^. The adsorption of organic molecules onto the metal surface would depend primarily on the surface charge of the metal and physicochemical properties of inhibitor molecules. Hence, such adsorption process was influenced by the presence of polar functional groups, steric factors, aromaticity, several heteroatoms (i.e. sulfur, nitrogen, oxygen), the electron density at the donor atoms, the orbital characteristics of the donating electrons, and the electronic structure of the inhibitor^16–20^. It has been believed that organic compounds were adsorbed onto the metal surface with an aid of their heteroatoms which possessed high basicity and electron density. They would work as corrosion inhibitor. In addition, these heteroatoms, which possessed the free electron pairs, would act as electron source for ‘electron transfer’. In these cases, donor groups were expected to facilitate efficient transfer of electrons and, also, lead to excellent adhesion performance during heterogeneous bonding system. Thus, a specific bond took place between unpaired electrons and coating surface. Organic compounds containing multiple bonds tend to be an effective inhibitor since the introduction of multiple bonds to chemical system should increase the inhibition efficiency of most organic compounds. If both the features of corrosion inhibitors and coating surface were combined, we believed that corrosion inhibition would be anticipated to be attained^16^.

The inhibitory roles of organic compounds, such as salicylaldoxime, thioacetamide, quinaldic acid, α-benzoionoxime, 2-(2-hydroxyphenyl) benzoxazole, cuprizone, and quinaldic acid, were understood in case of aluminum-based alloys^21–26^. The resilience of the passive alumina coating for long immersion time affected the formation of complex aluminium chelate. The inhibitory action of organic compounds during corrosion process occurred via the formation of protective film consisting of metal-organic complexes. Here, we considered diethyl-5-hydroxyisophthalate (DEIP) as kind of phenolic organic compounds would exhibit good efficiency of corrosion inhibition due to its hydroxyl (OH) and methoxy (OCH_3_) groups for succinct electron transfer.

The main purpose of the present work is to propose a concept of hybrid organic-inorganic coatings which has rarely been reported earlier. And, we look into a synergistic influence of inorganic coating with incorporation of TiO_2_ during PEO method and organic coating by organic inhibitor of DEIP during DC method on electrochemical stability of magnesium alloy. As to chemical analysis on a role of organic compound, quantum chemical calculations are used to reinforce theoretical rationale for the experimental results and to investigate the mechanism underlying the synergetic effect coming from hybrid organic-inorganic coatings in relation to electron transfer behaviour of DEIP.

## Results

### Surface morphologies of hybrid inorganic-organic coatings

**Figure 1:**
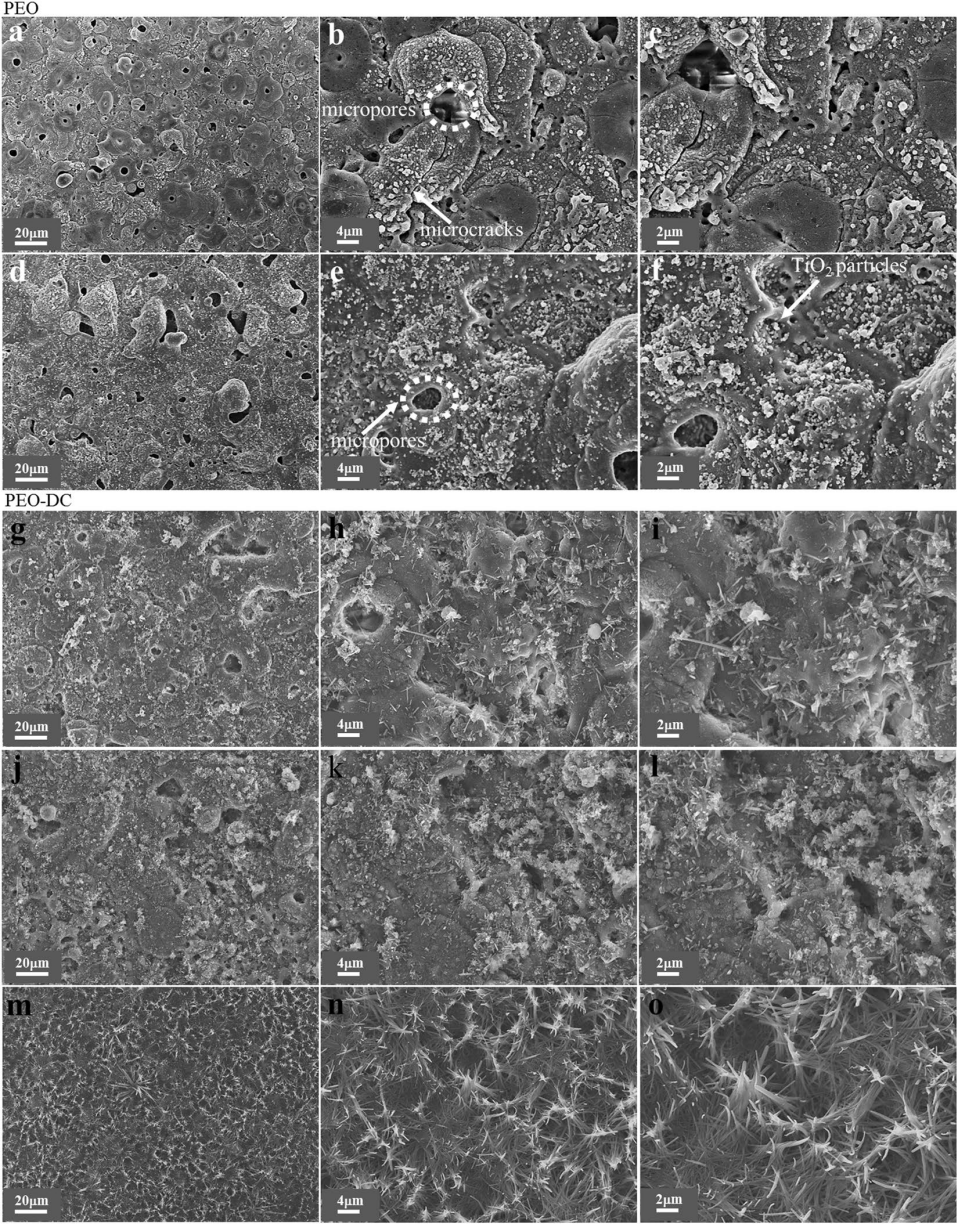
SEM images showing the surface morphologies of the coatings formed by using different methods: (**a–c**) coating layer formed by PEO process (sample I); (**d–f**) coating layer formed by PEO process with TiO_2_ particles (sample II); (**g–i**) coating layer formed by PEO process followed by DC in DEIP for 2 days at ambient temperature; (**j**–**l**) coating layer formed by PEO process with TiO_2_ particles followed by DC in DEIP for 1 day at ambient temperature; (**m**–**o**) coating layer formed by PEO process with TiO_2_ particles followed by DC in DEIP for 2 days at ambient temperature; figures are shown at various magnifications. Connected micropores were found in samples (I) and (II).

The surface morphologies of hybrid inorganic-organic coatings was illustrated in Fig. 1a–m; this process consisted of a two-step strategy involving plasma electrolytic oxidation for formation of the inorganic coatings and chemical treatment to load the surface with DEIP. Figure 1a–f reveals the differences in the surface morphology of the coatings generated via PEO with and without the addition of TiO_2_ particles. As shown in Fig. 1d–f, TiO_2_ particles were fused onto the surface of the inorganic coating and inside the open pores. In other words, the high magnification images of the samples with TiO_2_ (Fig. 1f) showed that TiO_2_ was present at the surface of the coating in the cavities (such as micropores). Although PEO with TiO_2_ resulted in more pores, their pore sizes were smaller than those obtained with the other coatings and a large number of the pores were partially filled. Therefore, the open pores on the coating surface were considered as uptake paths for particles to enter into the coating, given that the pores in the coatings deposited via PEO were partially filled with TiO_2_. Seyfoori *et al*.^25–29^ confirmed that the accumulation of particles in the vicinity of (and inside) the pores was higher than in the other zones. In general, the electrophoretic force of the particles, physical mixing, and vigorous fluctuation of the molten oxide by the plasma discharge are the main factors leading to the incorporation of particles into the inorganic coatings on Mg alloys^30, 31^. Moreover, the TiO_2_ particles incorporated into the inorganic coating might be either partly reactive or inert depending on the substrate, applied electrical parameters, electrolyte composition, and the properties of the particles^32^. However, TiO_2_ with a higher melting point would generally undergo inert incorporation or partly reactive incorporation, resulting in the formation of amorphous phases in the coatings^33^. The addition of TiO_2_ to the sodium aluminate electrolyte resulted in coverage of the sample surfaces (Fig. 1d–f) with a more uniform film than that shown in Fig. 1a–c ^32–34^. Therefore, the addition of TiO_2_ to the sodium aluminate electrolyte could induce significant changes in the surface morphology, producing a uniform film with less structural imperfections on the magnesium alloy.

The SEM images of all the coatings after 1 and 2 days of immersion in diethyl-5-ethylisophalate (DEIP) solution are presented in Fig. 1g–o. The organic compound was crystallized on the surface after 1 day of immersion. Figure 1g–l shows overall mono-phase precipitation of DEIP, whereas Fig. 1m–o shows bi-phasic DEIP precipitation after 2 days. Bulk DEIP adopted a needle-like shape on the inorganic coating surface without TiO_2_ (Fig. 1g–i), whereas the DEIP molecules on inorganic coating with TiO_2_ had a coral-like shape (Fig. 1j–l). Moreover, the sizes of the organic molecules on the AZ31 Mg alloy increased in the presence of the TiO_2_ particles, compared to the inorganic coating without particles. When the immersion time was increased to 2 days, DEIP covered almost the entire coating surface when the TiO_2_ particles were used. In other words, the amount of DEIP incorporated into the system increased with increasing immersion time. The facile formation of organic coating after immersion in DEIP solution might be due to the presence of the porous coating and the adsorbed functional groups on the TiO_2_ particles. Therefore, the adsorbed functional groups on the TiO_2_ surface significantly enhanced the nucleation of DEIP. In general, the solvent molecules (H_2_O and C_2_H_5_OH) could also be adsorbed on the Mg alloy or at the inorganic coating/ethanolic solution interface. Therefore, the adsorption of the organic compound from the ethanolic solution can be regarded as quasi-substitution between the organic molecules in the ethanolic solution [Org_(sol)_] and the water or ethanol molecules at the coating surface [H_2_O_(ads)_].

1 $$Or{g}_{(sol)}+x{H}_{2}O/{C}_{2}{H}_{5}O{H}_{(ads)}\leftrightarrow Or{g}_{(ads)}+x{H}_{2}O/{C}_{2}{H}_{5}O{H}_{(sol)}$$

In Eq. 1, *x* is the size ratio, i.e., the number of water or ethanol molecules replaced by one organic inhibitor molecule.

### Chemical composition of hybrid inorganic-organic coatings

**Figure 2:**
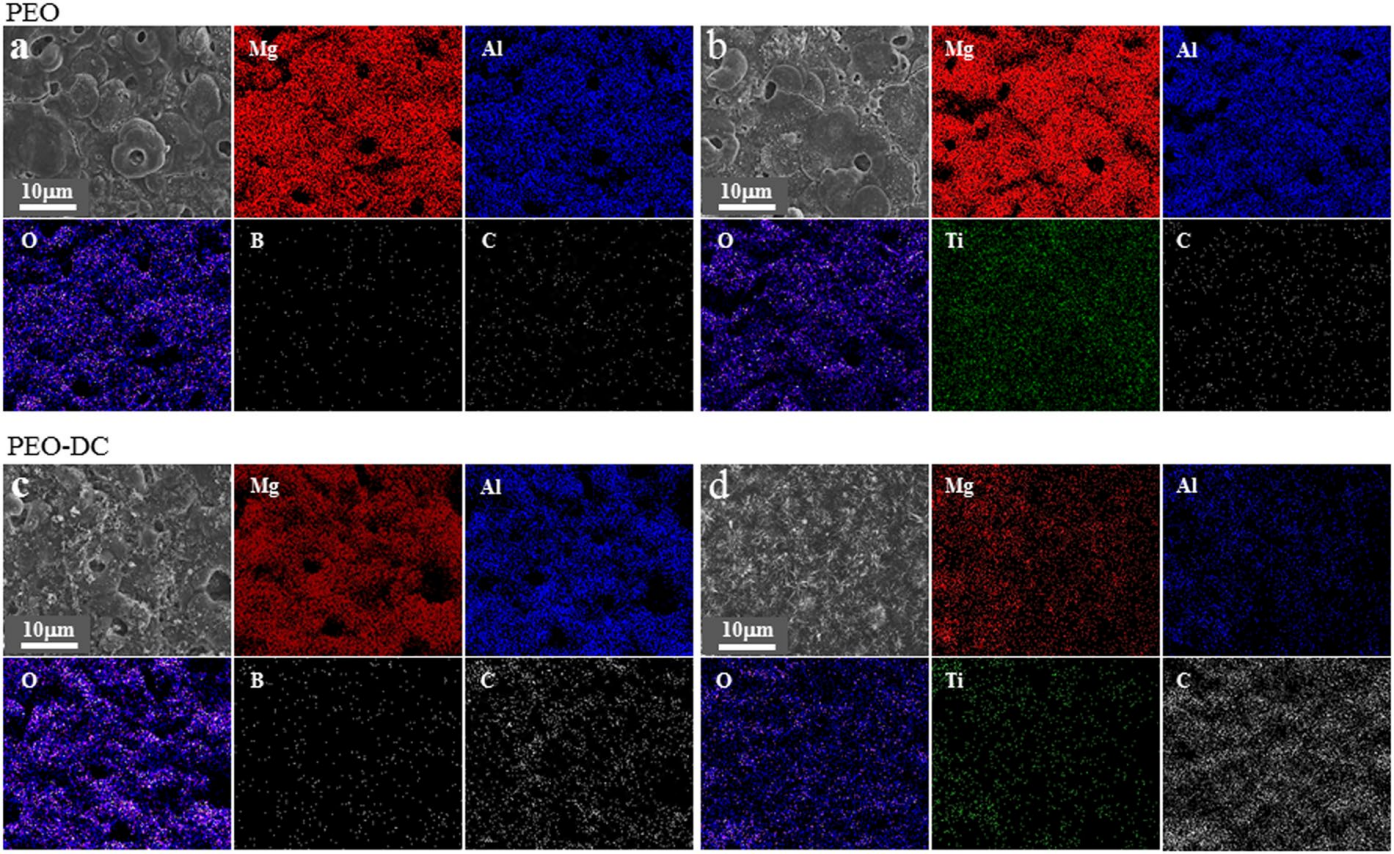
EDS mapping of the coating surface: (**a**) PEO coating (I); (**b**) PEO coatings with TiO_2_ particle (II); (**c**) PEO coating followed by immersion in DEIP solution for 2 days (III); (**d**) PEO coating with TiO_2_ particles followed by immersion in DEIP for 2 days (IV).

Figure 2 shows the elemental mapping images of Mg, Al, O, B, Ti, and C on the surface of the magnesium alloy without and with protection from the inorganic coating and organic coating. Carbon was not evenly distributed in over the unprotected magnesium alloy surface after immersion in ethanol. However, Mg, Ti, and Al were homogeneously distributed over the surface of the coated magnesium alloy, which suggests that corrosion would be effectively reduced by the synergistic effect. As shown in Fig. 2, EDS analysis indicated the presence of the characteristic elements on the coating surface. After modification of the surface with DEIP, the elemental composition changed. The contents of Mg, Al, and C (Table 1) in the coating that was not treated with DEIP were 33%, 15%, and 6%, respectively while the corresponding values of the samples treated with DEIP for 1 day were 24%, 9%, and 20% and those for the sample treated via PEO with TiO_2_ but not immersed in DEIP were 18%, 19%, and 2%. The corresponding values for the DEIP-treated coating were 8%, 2%, and 56%, respectively. The increase in the C content and the decreased Mg and Al content (shown in Table 1) were attributed to the aromatic structure of the organic compound formed on the coating surface. Considering the fact that the as-formed coating has endured rinsing from the ethanol solvent, it was indicated that dimethylene-5-hydroxyisophthalate film was successfully modified onto porous surface.

**Table 1:** Surface composition (at. %) of the coatings determined by EDS analysis.

Sample	Atomic ratio of Mg:Al:O:Ti:C				
	Mg	Al	O	Ti	C
I	33	15	45	—	6
II	18	19	44	16	2
III	24	9	47	—	20
IV	8	2	32	3	56

**Figure 3:**
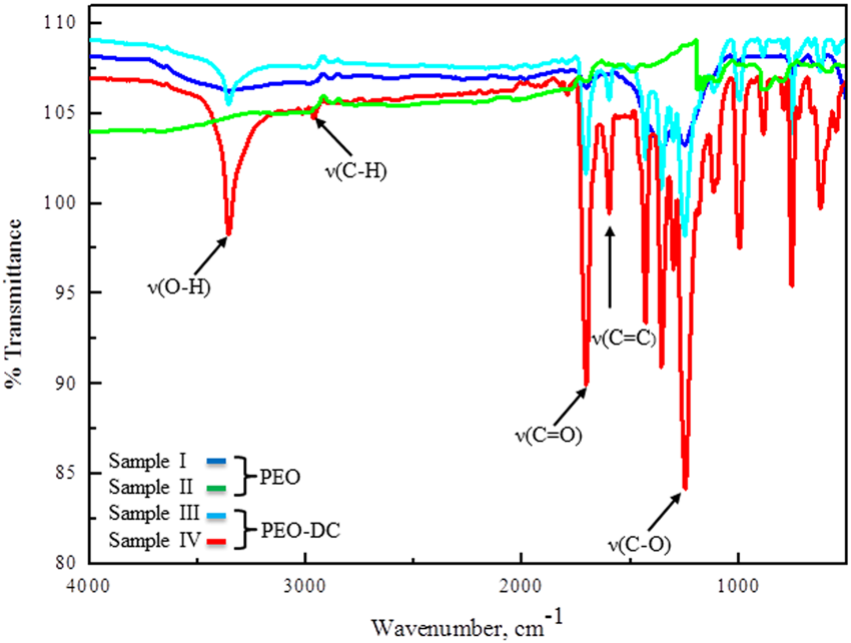
FT-IR data for coated materials with and without DEIP.

FT-IR spectra of the organic coating formed over the inorganic coating were taken at the range of 4000**-**400 cm^−1^ to analyse the functional groups of the organic coating after immersion in DEIP solution for up to 1 day (Fig. 3). FT-IR absorption bands were observed at 3361, 1706, 1596, 1432, 1356, 1238, 1115, 997, 883 and 756 cm^−1^. The broad band at 3361 cm^−1^ was attributed to the OH group and the band at 1706 cm^−1^ indicated the presence of C=O groups^35^. C-O stretching vibrations were observed at 1242 and 1115 cm^−1^, confirming the presence of phenolic and ester C-O in the organic coating^35^. The absorption bands at 988 cm^−1^ and 1090 cm^−1^ with a shoulder at 1119 cm^−1^ could be assigned to the ester group^36^. The bands at 1596 and 1432 cm^−1^ were attributed to the C=C stretching vibrations of the aromatic ring.

**Figure 4:**
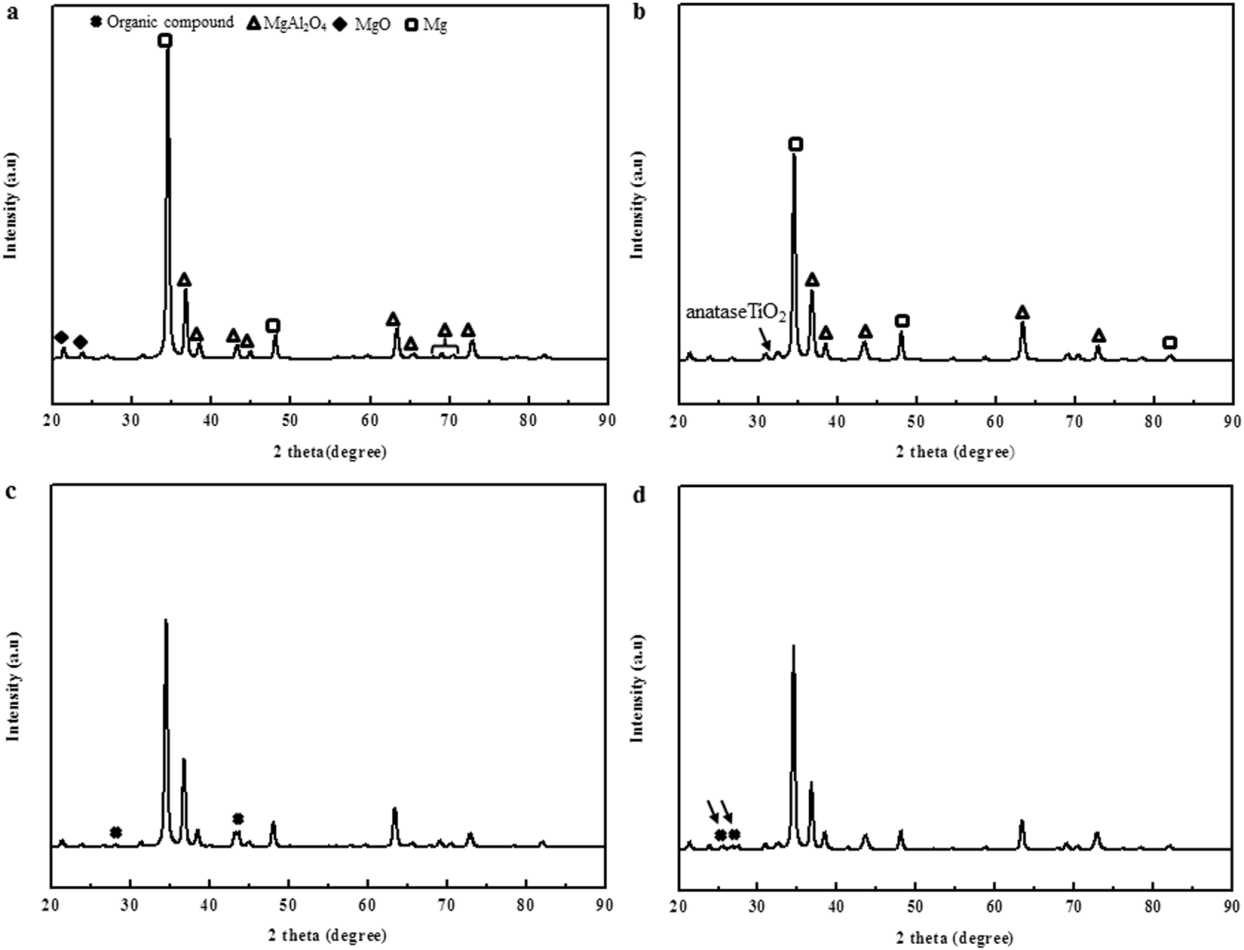
XRD analysis of the samples treated by various methods: (**a**) PEO coating, (**b**) PEO coating with TiO_2_ particles, (**c**) PEO coating followed by immersion in DEIP solution for 2 days, (**d**) PEO coatings with TiO_2_ particles, followed by immersion DEIP for 2 days. Scan range: 20°–90° with Cu-Kα radiation source.

As mentioned, XRD was used to determine the phase structure of the inorganic coating. Some peaks from the substrate were also observed in the spectrum. The phases of the organic coatings formed over the inorganic films containing anatase TiO_2_ after immersion in DEIP solution for 1 and 2 days were investigated by XRD (Fig. 4a–d). As shown in (Fig. 4c,d), characteristic diffraction peaks of the crystalline organic compound were clearly detected at 2θ = 26°, 27, 28°, and 44° in the XRD pattern, indicating that these needle- or coral-like species were assemblies of organic molecular crystals. After immersion, the surfaces were also partially covered with a discontinuous coating of molecular clusters. However, the intensity of the peaks of MgAl_2_O_4_, MgO, and Mg decreased after immersion in DEIP compared with the intensity of the peaks prior to immersion. This was further confirmed by changes in the relative intensities of the peaks corresponding to the organic compound (Fig. 4c,d). In comparison, the surfaces of the AZ31 Mg samples (Fig. 4a,b) were completely covered with a newly formed coating of molecular clusters, which exhibited flower-like morphology based on high magnification observation.

**Figure 5:**
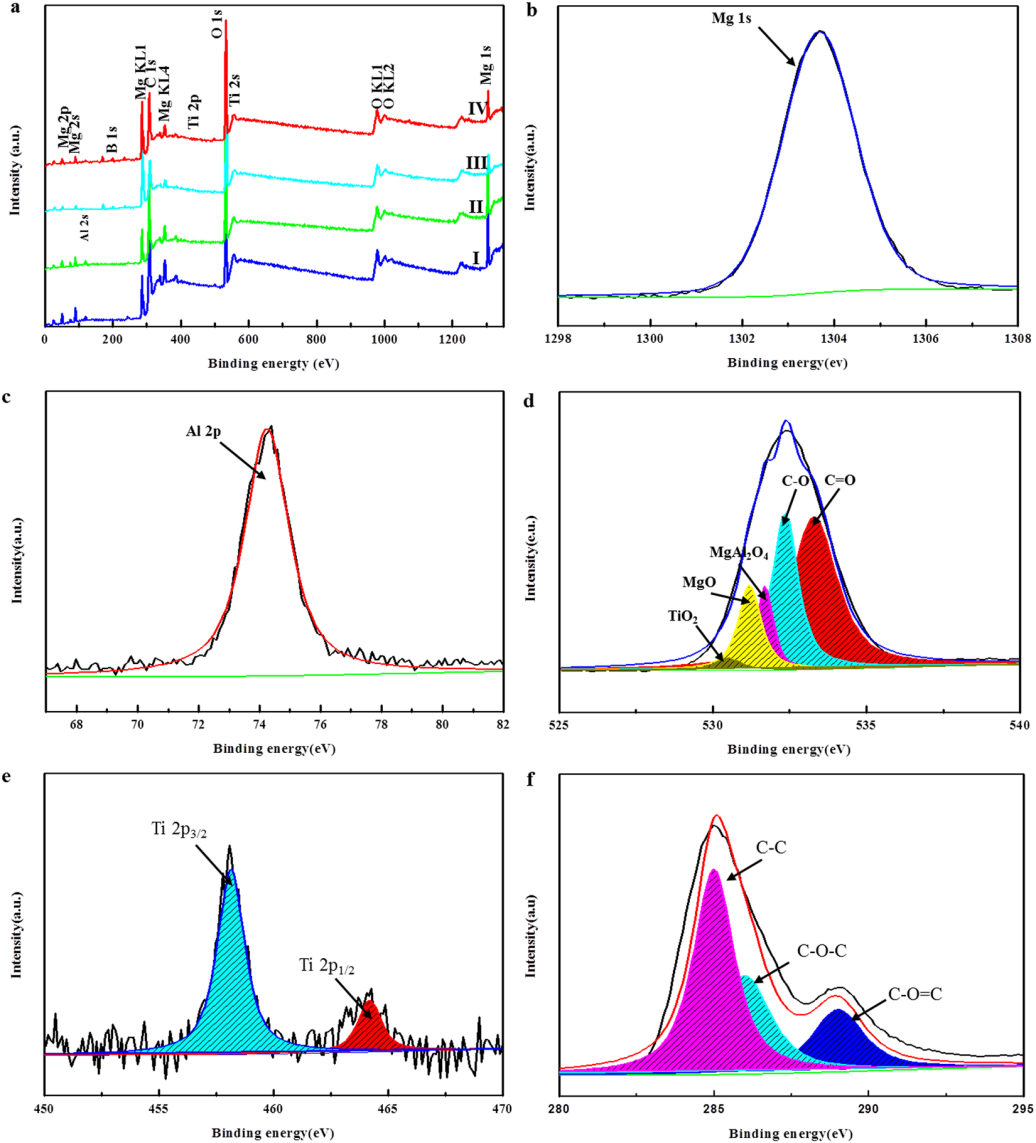
Full XPS spectra of (**a**) PEO coating (I); PEO coatings with TiO_2_ particles (II); PEO coating followed by immersion in DEIP solution for 2 days (III); and PEO coating with TiO_2_ particles followed by immersion in DEIP for 2 days (IV); corresponding high-resolution Mg 1 s (**b**), Al 2p (**c**), O 1 s (**d**), Ti 2p (**e**), and C (**f**) are presented.

From Fig. 5a, the hybrid organic-inorganic coatings consist of Mg, Al, Ti, B, O, K and C. The C was derived from the organic coating. The XPS O1s spectrum (in Fig. 5d) was wide and asymmetric, indicating the presence of more than one chemical state of O in the coating. After curve-fitting, four chemical states of surface O atoms were detected: O1s (Mg-O), O 1 s (MgAl_2_O_4_), O 1 s (C-O) and O 1 s (C=O) at about 532.33 ± 0.1, 531.7 ± 0.1, 531.21 ± 0.1, and 530.54 ± 0.1 eV, respectively. The symmetrical XPS Ti 2p spectra (Fig. 5e) could be deconvoluted into two peaks with binding energies of 458.18 ± 0.1 and 464.21 ± 0.1 eV, corresponding to Ti 2p_1/2_ and Ti 2p_3/2_, demonstrating that the main chemical state of Ti in the sample was +4^37^. As shown in Fig. 5c the Al 2p spectra displayed a single peak at 74.25 ± 0.1 eV, corresponding to MgAl_2_O_4_. The peak at 285 ± 0.1 eV in the C 1 s spectrum (Fig. 5f) was attributed to C-C/C-H groups from adventitious carbon. The other peaks around 286 ± 0.1 and 288.9 ± 0.1 eV corresponded to organic groups, indicating the presence of organic compound in the organic coating^27, 38, 39^. These might present because of the diffusion of organic compounds from the solution and their reaction with the inorganic coating. The Mg (2p_3/2_) (Fig. 5b) core level peak at a binding energy of 1303.68 ± 0.1 eV confirmed the formation of MgAl_2_O_4_^27, 40^. The O 1 s peak spread from 530 to 534.5 eV (Fig. 5d), which could be fitted to TiO_2_ at 530.54 ± 0.1 eV, MgO at 531.21 ± 0.1 eV, MgAl_2_O_4_ at 531.7 ± 0.1, C-O at 532.33 ± 0.1 and C=O at around 533.3 ± 0.1 eV. In summary, the organic and inorganic coatings of magnesium alloy were composed of TiO_2_, MgO, MgAl_2_O_4_, C-O, and C=O. Table 2 shows the atomic composition of the organic and inorganic coating. The amount of C increased and the content of Mg, Al, and Ti decreased after the immersion process, suggesting an increase in the content of the organic compounds in the organic coating on the treated samples. It would be noted that the C/Mg ratio of the coating increased from 3.79 to 15.3 after the AZ31 Mg treatment. This indicated that DEIP covered a high fraction of the inorganic surface coating.

**Table 2:** Atom percentages from XPS data for the sample coated the PEO process (I), PEO process with TiO_2_ particles (II), PEO process followed by immersion in DEIP solution for 2 days (III), and PEO process with TiO_2_ particles followed by immersion DEIP solution for 2 days (IV).

Sample	Element atom percentage (%)					
	Mg	Al	O	Ti	B	C
I	8.77	4.83	44.69	—	1.01	35.90
II	8.96	6.33	43.94	0.47	1.67	33.98
III	3.63	0.74	35.64	—	1.63	55.53
IV	4.88	1.66	37.53	0.22	2.32	50.20

### Electrochemical characterization of hybrid inorganic-organic coatings

**Figure 6:**
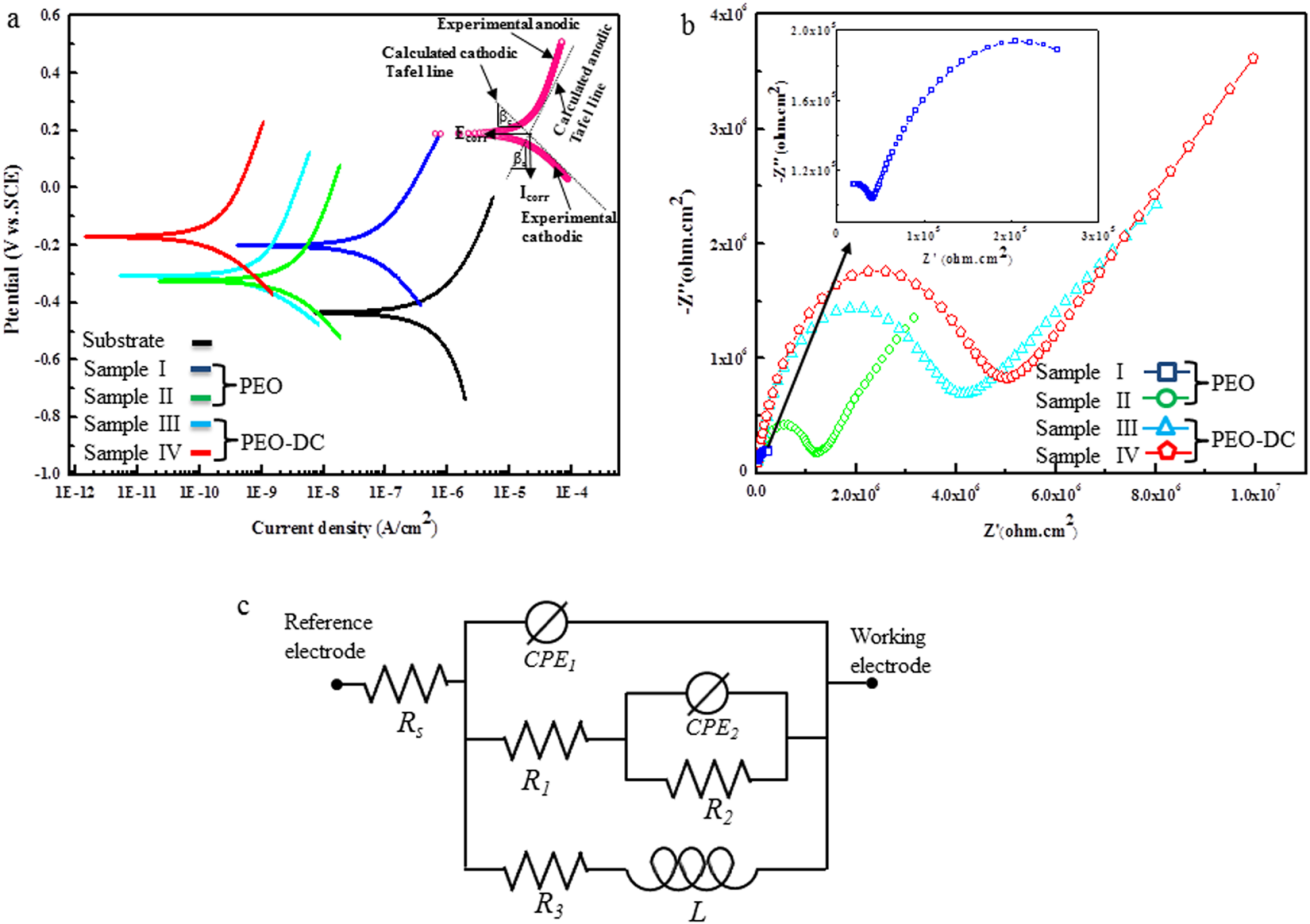
(**a**) Potentiodynamic polarization curves of the samples coated with PEO with and without TiO_2_ and DEIP; data were measured in the range of −0.3 to 0.4 V vs. open circuit potential in 3.5 wt.% NaCl solution. (**b**) EIS Nyquist plots of the samples coated via PEO with and without TiO_2_ and DEIP. (**c**) Equivalent circuit model consisting of the organic and inorganic coatings working as either resistors or condensers, and the solution resistance within the electrical cell.

The corrosion behavior of these coatings was evaluated by the potentiodynamic polarization technique in 3.5 wt.% NaCl solution at constant time, as shown in Fig. 6. The key electrochemical parameters, including the corrosion potential (*E*_*corr*_), corrosion current density (*i*_*corr*_), anodic and cathodic Tafel slope (*β*_*a*_, *β*_*c*_), and inhibition efficiency (η) derived from the polarization curves were given in Table 3. The values of η were calculated as follows^39, 40^.

2 $$\eta =\frac{{i}_{corr,0}-{i}_{corr}}{{i}_{corr,o}}\times 100$$

where *i*_*corr,o*_ and *i*_*corr*_ represent the current densities of the uncoated and coated magnesium samples, respectively.

**Table 3:** Potentiodynamic polarization analysis of the samples treated by various methods using different solutions; data were measured in the range of −0.3 to 0.4 vs. the open circuit potential in 3.5 wt% NaCl solution. *E*_*corr*_, *I*_*corr*_, *β*_*a*_ and *β*_*c*_ were obtained by extrapolations of the Tafel plots. η was calculated based on Eq. 1.

Sample	E_corr_ (mV)	i_corr_ (mV/cm^2^)	β_a_ (µ/cm^2^)	β_c_ (mV/cm^2^)	η (%)
substrate	−435	0.815	261 × 10^−3^	147 × 10^−3^	**—**
I	−202	9.63 × 10^−2^	436 × 10^−3^	333 × 10^−3^	88
II	−324	5.03 × 10^−3^	708 × 10^−3^	320 × 10^−3^	99.3
III	−307	1.17 × 10^−3^	605 × 10^−3^	191 × 10^−3^	99.8
IV	170	2.61 × 10^−4^	947 × 10^−3^	348 × 10^−3^	99.9

In the presence of DEIP (Fig. 6a), the polarization curves shifted to lower current densities, which indicated that the rate of corrosion of magnesium was suppressed to a certain extent, and the decrease in the cathodic current densities would be related to the reduction of dissolved oxygen^41^. These results revealed that this DEIP would act as corrosion inhibitor by inhibiting hydrogen reduction as well as magnesium anodic dissolution. In other words, it was clear that the protection from PEO coating alone was not sufficient to prevent severe corrosion of magnesium in NaCl solution. Therefore, as shown in Fig. 6a, corrosion resistance was obtained due to the synergetic action of PEO coating and organic coating than obtained with PEO coating alone. These results indicated that DEIP exhibited a favorable synergistic effect that prevents the corrosion of magnesium due to its adsorption and the formation of a protective film on the surface of the inorganic coating.

These organic-inorganic coatings caused a change in the anodic and cathodic Tafel slopes and no definite trend of the shift in the values with the use of different compositions of the inorganic coating could be observed, suggesting that these compositions behave as mixed-type (anodic/cathodic) inhibitors^42^. The hydrogen evolution was probably reduced by the surface blocking effect. Table 3 reveals that the corrosion current densities (*i*_*corr*_) decreased slightly with the PEO coating only. In contrast, the value of *i*_*corr*_ decreased obviously in the presence of both DEIP and PEO coating. These values of *i*_*corr*_ continued to decrease and η that was calculated from *i*_*corr*_ increased with the presence of DEIP. The maximum value of η (as shown Table 3) was 88% for the PEO coating (without TiO_2_ particles) but this value reached 99.9% due to the synergistic effect of DEIP with PEO coating (with TiO_2_ particles). It was thus evident that a more effective barrier film of inhibitor molecules was formed on the coating surface by synergy, thereby blocking the active sites on the magnesium surface to protect the magnesium alloy from corrosion^43–45^.

Figure 6b shows the impedance spectra obtained of hybrid inorganic-organic coatings after immersion in 3.5 wt.% NaCl in the absence and presence of the corrosion inhibitor at room temperature. The total impedance was higher for the system containing DEIP than for the system without DEIP, indicating that this inhibitor would protect the magnesium alloy against corrosion. These results were confirmed by the corresponding impedance spectra, where higher impedance was observed for the systems containing DEIP (from the semicircles at low frequencies) compared to that of the system without the inhibitor. This difference indicated that the inhibitor protects the magnesium alloys from corrosion (Fig. 6b). The Nyquist spectra had two characteristic capacitive loops in the high and low frequency ranges, respectively^46^. Figure 6c presents the equivalent circuit model assigned for all of the present samples, from which the parameter values were obtained and listed in Table 4. The model used to explain the corrosion behavior of the Mg alloy was based on the impedance plots and EIS studies of Gao *et al*.^47^, Baril *et al*.^48, 49^, and Song *et al*.^50^ on the PEO coating. In the circuit, *R*_*s*_ represents the corrosion resistance of the NaCl solution between the sample and counter electrode; *R*_*1*_ is the outer part resistance, and CPE_1_ was the constant phase element, which was related to the capacitance of the organic coating; *R*_*2*_ represents the contribution of inner part to the overall corrosion resistance (Fig. 6c) and the corresponding capacitance was represented by CPE_2_. *R*_*ct*_ and *L* are in series to represent the inductive impedance behavior, where *R*_*ct*_ represents the charge transfer resistance and *L* indicated the corrosion behavior at low frequencies^51, 52^. Table 4 shows the EIS fitting data obtained from the equivalent circuits and the experimental values obtained from the impedance data.

**Table 4:** Electrochemical impedance parameters for the samples subjected to PEO tratment using different electrolytes. All values are iterated by the analysis in the context of the equivalent circuit model.

Sample	R_1_	R_2_	R_ct_	CPE1-T/S.s^n^	CPE1-P	CPE2-T/S.s^n^	CPE2-P	L/H cm^−2^
I	4.53 × 10^+4^	2.91 × 10^+6^	4.35 × 10^+5^	1.40 × 10^−6^	0.64	3.29 × 10^+9^	0.67	3.25 × 10^−4^
II	1.09 × 10^+6^	6.56 × 10^+8^	1.86 × 10^+7^	2.82 × 10^−7^	0.41	1.65 × 10^−10^	0.81	2.68 × 10^+5^
III	3.68 × 10^+6^	2.55 × 10^+9^	5.54 × 10^+7^	1.45 × 10^−7^	0.33	1.47 × 10^−10^	0.83	1.01 × 10^+3^
IV	4.63 × 10^+6^	4.61 × 10^+9^	9.14 × 10^+7^	1.17 × 10^−7^	0.39	1.40 × 10^−10^	0.82	1.64 × 10^+3^

### Quantum chemical calculation

**Figure 7:**
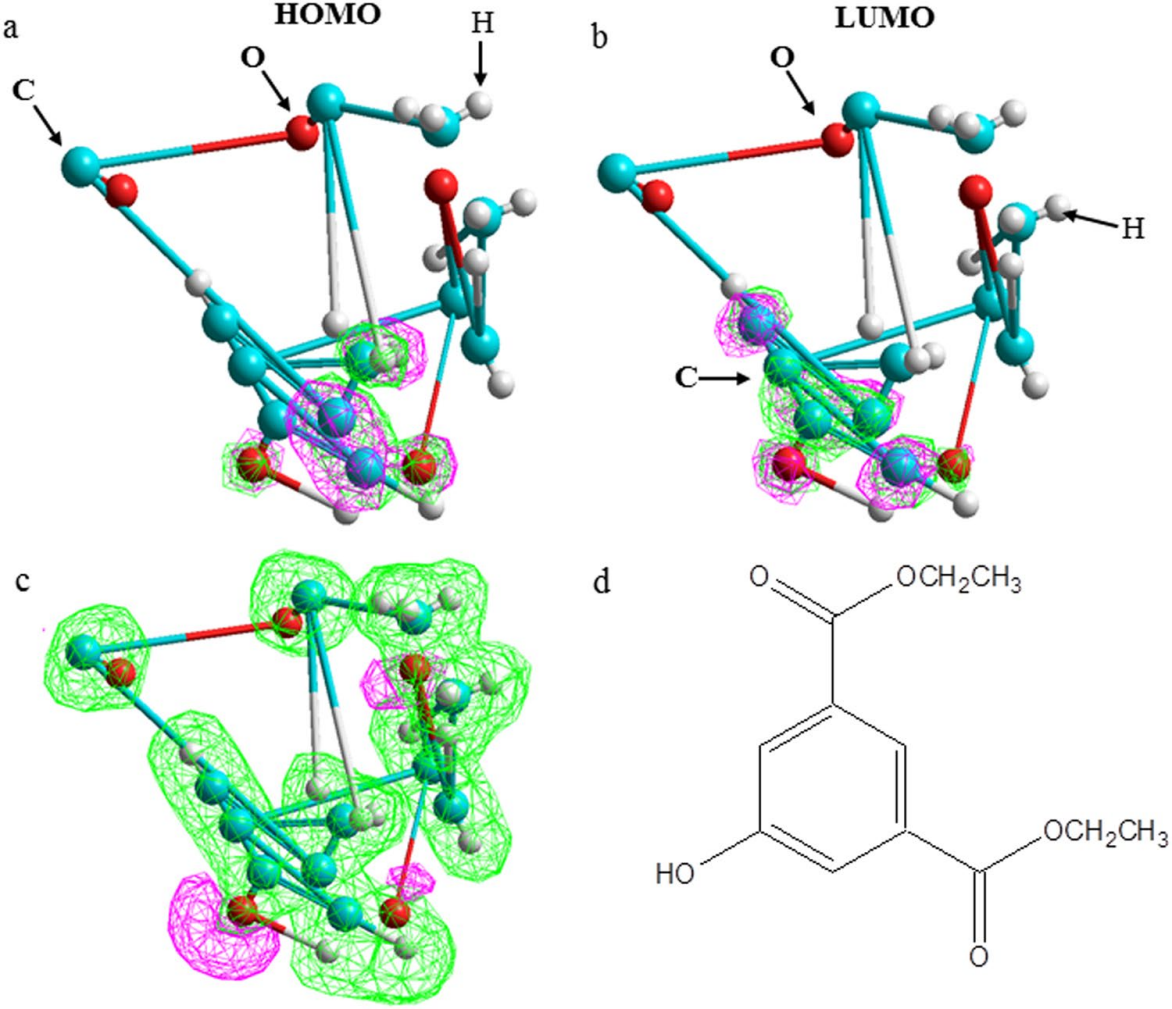
(**a**) Highest occupied molecular orbitals (HOMO), (**b**) lowest unoccupied molecular (LUMO), and (**c**) molecular electrostatic potential map (MP) of (**d**) structural of diethyl-5-hydroxyisophalate (DEIP).

Quantum chemical calculations have been used to study the relationship between the molecular/electronic structure of an organic inhibitor and its inhibition properties. Figure 7 shows the equilibrium geometry structure, frontier molecular orbital density distribution, the neutral form, and electrostatic potential (ESP) map of the organic molecule. The obtained quantum chemical parameters, such as the energy of the highest occupied molecular orbital (*E*_*HOMO*_), the energy of the lowest unoccupied molecular orbital (*E*_*LUMO*_), separation energy (*ΔE*), dipole moment (*μ*), electronegativity (*X*) and fraction of electron transfer (*∆N*) for the DEIP molecule are listed in Table 5. It was known that *E*_*LUMO*_ was related to the electron accepting ability of a molecule, whereas *E*_*HOMO*_ was often associated with the capacity of a molecule to donate electrons. In other words, a high value of *E*_*HOMO*_ was likely to indicate a tendency of the molecule to donate electrons to appropriate acceptor molecules with low energy and empty molecular orbitals, whereas the lower the value of *E*_*LUMO*_, the more probable it is that the molecule will accept electrons^53, 54^. However, the *∆E* described the stability of a molecule; smaller values of the separation energy (energy gap) (*E*_*LUMO*_-*E*_*HOMO*_, *∆E*) indicated that a molecule would potentially be adsorbed more easily on the surface of the inorganic coating. As the energy gap decreased, the reactivity of the molecule towards the inorganic coating increases, thus leading to enhanced of the inhibition efficiency of the molecule. Additionally, the dipole moment (*µ*) has been widely used to indicate the polarity of inhibitor molecule. Higher values of *μ* correspond to enhanced corrosion inhibitor molecule^55, 56^. Indeed, the dipole moment of DEIP is 2. 2.768 D, which was higher than that of a water molecule (i.e., 1.88 D). The high dipole moment probably increases the adsorption between the corrosion inhibitor and coating surface. These results in adsorption of DEIP on the surface of the inorganic coating, accompanied by desorption of water molecules, thereby protecting the magnesium alloy against the NaCl solution. Furthermore, according to Koopmans’ theorem^57^, the frontier molecular orbital energies *E*_*HOMO*_ and *E*_*LUMO*_ were related to the ionization potential (*I*) and the electron affinity (*A*) of the magnesium atoms and the inhibitor molecules.

3 $${\rm{I}}=-{{\rm{E}}}_{{\rm{HOMO}}}\,{\rm{and}}\,{\rm{A}}=-{{\rm{E}}}_{{\rm{LUMO}}}$$

**Table 5:** Quantum chemical parameters for diethyl-5-hydroxyisophalate (DEIP).

Inhibitor	E_HOMO_(eV)	E_LUMO_(eV)	∆E(eV)	μ(Debye)	A	I	X(eV)	γ(eV)	∆N
DEIP	−9.473	−0.135	9.337	2.768	0.135	9.473	4.804	4.668	−0.09
Mg	−7.661	1.717	9.378	1.310	−1.717	7.661	2.972	4.689	

The absolute electronegativity (*X*) and the global hardness (*γ*) of the inhibitor molecule can be described as follows^58^.

4 $${\rm{X}}=\frac{{\rm{I}}+{\rm{A}}}{2}$$

5 $$\gamma =\frac{{\rm{I}}-{\rm{A}}}{2}$$

Finally, the fraction of electrons transferred from the inhibitor to the metallic surface, *∆N*, is given by Eq. 6 ^59^.

6 $${\rm{\Delta }}N=\frac{{{\rm{X}}}_{{\rm{Mg}}}-{{\rm{X}}}_{{\rm{inh}}}}{2({{\rm{\gamma }}}_{{\rm{Mg}}}+{{\rm{\gamma }}}_{{\rm{inh}}})}$$

Thus, to calculate the fraction of electrons transferred (*∆N*), the theoretical value of the electronegativity of the Mg atom, *X*_*Mg*_(2.972 eV) and the global hardness of the Mg atom, γ_Mg_, (4.689 eV) were employed. *X*_*inh*_ and *γ*_*inh*_ are the absolute electronegativity and global hardness of the DEIP molecules, respectively. Indeed, the *∆N* values were correlated to the inhibition efficiency resulting from electron donation. If *∆N* < 3.6 (Table 5), the inhibition efficiency would be increased with increasing electron-donation ability to the metal surface^60, 61^.

As seen from Fig. 7, the preferred active site for electronic attack and the favorited sites for interaction with the metal surface were located within the region close to the O atoms and aromatic ring. In addition, the electrons of the O atoms on the polar groups were completely transferred to the empty d or p orbitals of the magnesium atom.

## Discussion

### Electrochemical behaviour

In the presence of DEIP, potentiodynamic polarization results in a 3.5 wt.% NaCl showed an appreciable decrement in both anodic and cathodic current densities. The corrosion current decreased with an increase in the concentration of EDIP on the surface due blocking of the active sites of the inorganic coating^62^. The addition of DEIP has an inhibitive effect on both the anodic and cathodic parts of the polarization curves at lower current densities. This result suggested that the addition of DEIP would be reduced the dissolution of the anode and retards the hydrogen evolution reaction (Table 3). Furthermore, addition of DEIP induced a small positive shift of *E*_*corr*_ values, suggesting that DEIP would act as a mixed type inhibitor, predominantly inhibiting the anodic reaction^63^. The variations of *β*_*c*_ and *β*_*a*_ showed that both the cathodic and the anodic reactions of the corrosion process were suppressed by the organic and inorganic coating on the surface of the magnesium alloy. For a comprehensive investigation on the corrosion behaviour, EIS was also performed in a 3.5 wt.% NaCl. By fitting these curves to a suitable equivalent circuit shown in Fig. 6c, the corrosion parameters were determined and recorded in Table 4. Assessment of Table 4 shows that the addition of DEIP increased the *R*_*1*_ and *R*_*2*_ values, and the effect was enhanced with increasing concentration. Thus, it would be inferred that DEIP adsorption layers were formed on the metal surface, indicating the adsorption of this compound on the surface of AZ31 Mg and more efficient blocking of the surface against charge and mass transfer^63–65^. As shown in Table 4, the values of the outer part resistance increased with an increase in the coating thickness. This increase could be attributed to the formation of an organic coating on the surface of the inorganic coating. Furthermore, it was evident that both DEIP and TiO_2_ exhibited a synergistic effect that led to higher resistance of all parts of the coating layers. On the other hand, the CPE decreased with an increase in the coating thickness, which can be attributed to enhancement of the thickness of the electrical double layer^66^. The coating layers between the charged metal surface and the solution can be considered as an electrical capacitor^65, 66^. In addition, the charge transfer resistance (Table 4) increased with an increase in the concentration of the inhibitor. This was very important because higher *R*_*ct*_ values indicated better inhibition performance.

### Mechanism of hybrid inorganic-organic coatings

**Figure 8:**
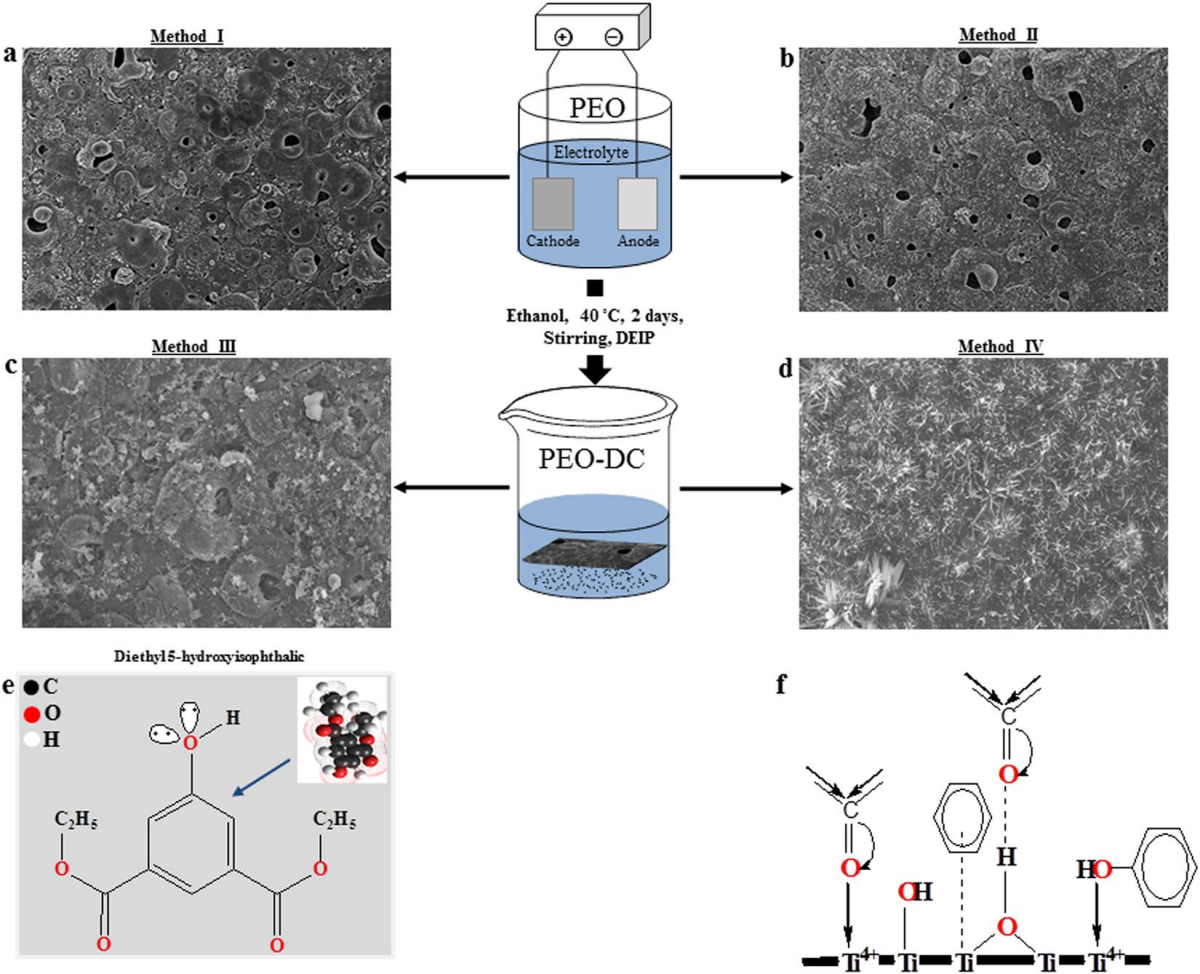
Schematic illustration of the fabrication process for the samples, including plasma electrolytic oxidation (PEO) and chemical treatment (DC) with a corrosion inhibitor. (**a**) coating layer formed by PEO process (I); (**b**) coating layer formed by PEO process with TiO_2_ particles (II); (**c**) coating layer formed by PEO process followed by DC in DEIP for 2 days at ambient temperature (III); (**d**) coating layer formed by PEO process with TiO_2_ particles followed by DC in DEIP for 2 and days at ambient temperature (IV); (**e**) chemical structure of the investigated inhibitor; (**f**) the adsorption sites of DEIP which adhere with inorganic coating in the present of TiO_2_.

Figure 8 shows the schematic illustration describing the fabrication process of the hybrid organic-inorganic coating via PEO and chemical DC. During the PEO, the main electrochemical reactions occurring at the coating/electrolyte interface using the aluminate-containing electrolyte are as follows:

During the PEO process, Mg would transform into Mg (II) according to by the reaction:

7 $$Mg\to M{g}^{2+}+2e$$

These ions would be combined with other anions such as AlO_2_^−^ and OH^−^ in discharge channels; these anions were obtained from the components of the electrolyte.

8 $$M{g}^{2+}+2Al{O}_{2}^{-}\to MgA{l}_{2}{O}_{4}\downarrow $$

9 $$M{g}^{2+}+2O{H}^{-}\to Mg{(OH)}_{2}$$

As a result of the thermal energy from the discharges, the magnesium hydroxide Mg(OH)_2_ was converted to magnesium oxide (MgO) by the dehydration process in Eq. 10 ^67^.

10 $$Mg{(OH)}_{2}\to MgO\downarrow +{H}_{2}O$$

MgO and MgAl_2_O_4_ were the main phases formed during coating in the aluminate based electrolyte (Fig. 4). Duan *et al*.^68^ also investigated magnesium coating in several electrolytes with different additives and observed various phases. Based on previous reactions, in the present case with and without TiO_2_, the pores arise at the sites of the discharge channels due to gas release (mainly O_2_ and H_2_) through the molten inorganic materials^27, 69^. Moreover, some micro-cracks (Fig. 8a,b) were formed on coating surface due to thermal stress and high-energy discharge during the oxidation process. The incorporation mechanism of the particles into PEO coatings on the AZ31 Mg alloy was reported by Al Zoubi and coworkers^27^.

Under the strong electric field of PEO, the metal cations that were transferred from magnesium alloy to react with the anions to form a porous inorganic coating. It was well recognized that the second step in the AZ31 Mg alloy coating process was the adsorption of DEIP at the inorganic layer/solution interface and that the adsorption depends on the molecule’s chemical composition, the temperature, and the electrochemical potential at the inorganic layer/solution interface. The presence of DEIP structure as organic coating would be confirmed by the FT-IR spectra data presented in Fig. 3, which showed the absorption bands of hydroxyl group in the region of 3361 cm^−1^ and the carbonyl group in the region 1706 cm^−1^. Moreover, the EDS data also indicated that organic coating was present on the inorganic coating, which was confirmed by the XRD results (Fig. 4).

The growth mechanism of the organic coating during the immersion process was presented in Fig. 8c,d. The adsorption of the inhibitor on the coating surface would be influenced by several factors, including the chemical structure of the inhibitor, the distribution of charge over the entire inhibitor molecule (Fig. 8e,f) and the nature and charged surface of the inorganic coating^70^. The adsorption of DEIP on the coating surface occurs by several ways: (a) electrostatic interaction between the charged centres (Fig. 8e,f) of the molecules and the charged coating, (b) donor-acceptor interaction between unshared electron pairs (Fig. 8) in the molecule and the vacant d-orbital of the metal surface atoms, (c) donor-acceptor interaction between the π-electrons of the aromatic ring and the multiple bonds and vacant d-orbitals of the metal surface atoms (Fig. 8e,f and d) donor-acceptor interaction between the d-electrons of surface metal-atoms (Mg, Al and Ti) (Fig. 8f) and the empty antibonding molecular orbital of DEIP (retro-donation)^69^. It was particularly significant that the inhibition efficiency afforded by the organic compound may be attributed to the presence of electron rich O and aromatic rings. DEIP contains one aromatic ring as well as ester and hydroxyl groups. Thus, the possible reaction centres was unshared electron pairs of the heteroatoms and π-electrons of the aromatic ring. In general, in ethanolic solutions, the organic molecules exist either as neutral molecules (phenol) or in the form of protonated organic molecules (phenolate ion or phenoxide ion) (Fig. 8b).

Based on the proposed mechanism, the neutral organic compound might be adsorbed on the surface of the inorganic coating via the chemisorption mechanism (Fig. 8f), involving the displacement of water or ethanol molecules from the surface coating or metal (through the pores) (Eq. 1) and the sharing of electrons between the hetero-atoms and metals^71^. The adsorption of organic molecules from the ethanolic solution onto the coating surface containing TiO_2_ proceeded without reaction products. The amount adsorbed increased steeply with an increase in the immersion time. Figure 8(c,d) shows the surface morphologies of the prepared inorganic coating after immersion in DEIP for 2 days. When the immersion time was increased to 2 days, the organic coating covered uniformly the inorganic coating containing TiO_2_. The facile formation of the organic coating after immersion in DEIP solution might be due to the presence of the TiO_2_ particles and the porous inorganic coating. A comparison of the SEM images micrographs (Fig. 8) of the magnesium alloys in the presence of the corrosion inhibitor utilized in this study with those of the uninhibited surface confirmed that the inhibitor compound was adsorbed on the surface of the inorganic coating. The inorganic coating containing the TiO_2_ particles offers the advantage is the formation of strong van der Waals bonds between the organic molecules and the inorganic coating surface. The TiO_2_ particles in the inorganic coating increased the density, flexibility, and functional compatibility with the organic coating. However, the use of TiO_2_ particles can improve the bonding to specific organic compounds (Fig. 8) in comparison to inorganic materials based on magnesium oxide and magnesium aluminates.

## Conclusions

A novel comprehensive method was proposed in this study to develop the heterogeneous multi-layer which resulted from the growth of DEIP organic coating on top of inorganic coating by plasma electrolytic oxidation in the electrolyte containing TiO_2_ particles. The empirical and theoretical finding presented in this study led to the following points:

1. During PEO, the inert incorporation of TiO_2_ nanoparticles was useful enough to seal the micro-defects in the inorganic coating, giving rise to the improved corrosion resistance and the formation of organic coating. During DC, the physical bonds were generated over the entire inorganic coating, which significantly enhanced the growth of hybrid organic-inorganic coating. According to FT-IR spectra, hydroxyl group at a region of 3361 cm^−1^ in organic coating played a key role in determining chemical and physical adsorptions onto inorganic coating incorporating TiO_2_.
2. As to formation mechanism of hybrid organic-inorganic coatings, the organic compounds might be adsorbed on the surface of inorganic coating via chemisorption mechanism, involving the displacement of polar molecules from the surface coating and the electron transfer between the heteroatoms and inorganic coating. Based on quantum chemical prediction, the values of *E*_*HOMO*_, *E*_*LOMO*_, *∆N*, electronegativity *(X)*, and the dipole moment (*µ*) would support the experimental results, providing deep insight into the adhesion mechanism of hybrid organic-inorganic coatings.
3. The electrochemical analyses using potentiodynamic polarization and impedance spectroscopy tests in 3.5 wt% NaCl solution confirmed that excellent corrosion resistance would be attained by hybrid organic-inorganic coatings. The use of DEIP resulted in excellent efficiency to inhibit corrosion by preventing anodic magnesium dissolution as well as cathodic hydrogen evolution.

## Materials and Methods

### PEO coating

An AZ31 magnesium alloy plate comprising Al, Zn, Mn, Fe, and Si in ratios of 2.89, 0.96, 0.31, 0.15, and 0.12 wt.% (balance Mg) was cut into samples with dimensions of 20 mm (L) × 20 mm (W) × 4 mm (T) for use as the working electrode (anode) in these experiments. All samples were mechanically ground with SiC paper up to 1200 grit, rinsed with distilled water and cleaned ultrasonically with pure ethanol prior to PEO coating. The present samples were set as the anode and a stainless steel net was used as the cathode. A glass vessel was used as the electrolyte container, equipped with a magnetic stirrer and a water cooling system to maintain the electrolyte temperature around 288 K for stabilization of the electrochemical reactions throughout the process. The PEO coating treatments were performed for 5 min under an AC (alternative current) power supply (100 mAcm^−2^) with a frequency of 50 Hz. The following reagent-grade chemicals were used to prepare the electrolytes: potassium hydroxide (3 g L^−1^ KOH), sodium aluminate (8 g L^−1^ NaAlO_2_), sodium tetraborate decahydrate (5 g L Na_2_B_4_O_7_.10H_2_O), titanium dioxide (5 g L^−1^ TiO_2_), glycerol (6 g L^−1^ C_3_H_8_O_3_) and hexamethylenetetramine (5 g L^−1^ (CH_2_)_6_N_4_) (Table 6). For the electrolyte, the pH and electric conductivity were approximately ~13 and ~19 mScm^−1^, respectively. All the chemicals used herein were purchased from Aldrich or Merck unless otherwise cited.

**Table 6:** Composition of the solutions and the methods used for the inorganic and organic coatings.

Sample	Method	NaAlO_2_	KOH	Na_2_B_4_O_7_	TiO_2_	C_3_H_8_O_3_
I	PEO coating	8	4	5	—	4
II	PEO coating with TiO_2_	8	4	5	5	4
III	PEO coating after immersion	C_10_H_10_O_5_				
IV	PEO coating with TiO_2_ after immersion	C_10_H_10_O_5_				

### Dip-coating as post-treatment

For dip-coating with the organic compound (Table 6), the PEO-coated samples were immersed in an ethanolic solution of 1 M DEIP for 1 day and 2 days, respectively, at 298 K, with consecutive drying and air annealing in a muffle furnace at 373 K for 1 h.

### Electrochemical assessment

To evaluate the corrosion behavior of the coating layers, potentiodynamic polarization measurements were carried out in a 3.5 wt.% NaCl solution at 25 °C using a three-electrode system with a ZAHNER Im6ex (Germany) electrochemical workstation. The electrochemical measurements were carried out in a potentiostat (Gamry Interface1000) with a three-electrode cell system. The PEO-coated sample was used as the working electrode (WE), platinum (1 cm^2^ area) was used as the reference electrode (SCE). The potentiodynamic polarization data were acquired in the range of −0.3 V to 0.4 V with respect to the open circuit potential (OCP) at scan rate of 1 mVs^−1^ and the EIS experiments were conducted from 10^6^ to 0.1 Hz at an interval of 10points/decade with 10 mV rms. The corrosion current density (*I*_*corr*_) and corrosion potential (*E*_*corr*_) values were obtained using the Tafel extrapolation method, and the inhibition efficiency η (%) was calculated by using Eq. 11.

11 $${\rm{\eta }}\, \% =\frac{{{\rm{I}}}_{{\rm{corr}}}^{0}-{{\rm{I}}}_{{\rm{corr}}}}{{{\rm{I}}}_{{\rm{corr}}}^{0}}\times 100$$

where ${I}_{corr}^{0}$ and ${I}_{corr}$ are the corrosion current densities in the absence and presence of the coating layer, respectively.

### Quantum calculation

Hyper Chem-8 was a sophisticated molecular modeler, editor, and powerful computational package that was known for its quality, flexibility, and ease of use. This program combines 2D visualization and animation with quantum chemical calculations, molecular mechanics, and dynamics. In the present work, the parameterization method 3(PM3) was used for calculation of the heat of formation and binding energy for all metal complexes. PM3 was more widely recognized than other semi-empirical methods due to the availability of algorithms and greater accuracy compared with other methods^72^. It was parameterized primarily for organic molecules and selected metals.
